# Sleep-disordered breathing and asthma: evidence from a large multicentric epidemiological study in China

**DOI:** 10.1186/s12931-015-0215-5

**Published:** 2015-05-10

**Authors:** Liwen Li, Zhiwei Xu, Xingming Jin, Chonghuai Yan, Fan Jiang, Shilu Tong, Xiaoming Shen, Shenghui Li

**Affiliations:** Xin Hua Hospital, School of Medicine, Shanghai Jiaotong University, Shanghai, China; School of Public Health, Shanghai Jiao Tong University School of Medicine, 227 South Chongqing Road, Shanghai, 200127 PR China; School of Public Health and Social Work, Queensland University of Technology, Brisbane, Qld Australia; Children’s Medical Center, School of Medicine, Shanghai Jiaotong University, Shanghai, China; MOE - Shanghai Key Laboratory of Children’s Environmental Health, Xinhua Hospital, Shanghai Jiao Tong University School of Medicine, 1665 Kong Jiang Road, Shanghai, 200092 China

**Keywords:** Children, Asthma, Sleep-disordered breathing, Cross-sectional, Meta-analysis

## Abstract

**Background:**

Previous studies have postulated that sleep-disordered breathing (SDB) may be associated with the occurrence and exacerbation of asthma. However, there was limited quantitative evidence on the topic. This study aimed at investigating the prevalence and predisposing factors of asthma, and quantifying the association between SDB and asthma among school-aged children in China. In addition, a comprehensive meta-analysis of the published evidences and our findings were further conducted.

**Methods:**

To test the hypothesis, we conducted a multicentric cross-sectional study involving 22,478 children aged 5–12 years recruited from eight cities in China. Furthermore, a meta-analysis based on both previously published studies and our cross-sectional study was performed.

**Results:**

The prevalence rate of SDB and asthma was 12.0% and 3.5% among our cross-sectional study sample. It was demonstrated that symptoms of SDB, such as habitual snoring (OR = 1.28, 95%CI: 1.01-1.62), and obstructive sleep apnea (OSA) (OR = 1.92, 95%CI: 1.34-2.76), were significantly associated with asthma, after adjusting for potential confounding factors. In the meta-analysis, SDB was correlated with the prevalence of asthma in both children (OR = 1.58, 95%CI: 1.35-1.80) and adults (OR = 1.55, 95%CI: 1.42-1.67).

**Conclusions:**

Our results provide further evidence for the independent association between SDB and asthma. The clinical significance of our findings lies in the emphasis that children undergoing examination or treatment for asthma should be routinely screened for sleep problems. Further systematic study is required to illuminate the underlying mechanism.

## Introduction

Asthma is one of the primary causes of chronic respiratory disorders [1]. Over the last quarter century, a dramatic increase in the morbidity and economic burden of asthma has become a public concern [2]. It was reported that at least 5% of the general population suffered from asthma [3,4]. Obesity is a well-established risk factor for asthma in adults, but the mechanism underlying the association is still far from clear [5]. Epidemiological studies suggested that obesity and obesity related co-morbidities, including sleep-disordered breathing (SDB) and gastro-oesophageal reflux (GOR), were highly prevalent in children with asthma [6-8].

SDB and asthma share similar diurnal and nocturnal symptoms. Airway obstruction is involved in the pathogenesis of both diseases [9,10]. The term “SDB” refers to a spectrum of abnormal breathing and/or gas exchange patterns during sleep. It is characterized by habitual loud snoring and increased respiratory effort. Habitual snoring, upper airway resistance syndrome (UARS), obesity hypoventilation, obstructive sleep apnea (OSA), and central sleep apnea are the primary syndromes that fall under the rubric of SDB [11]. The first report regarding the significant association between habitual snoring and history of exercise-induced asthma can date back to 1992 [12]. Since then, several studies have been conducted to explore the correlation between sleep disturbance and asthma in SDB patients. [13-17]. However, few studies investigated the prevalence of SDB in asthma patients. To our knowledge, so far, only three, two cross-sectional and one cohort, studies examined the prevalence of SDB in children with a confirmed diagnosis of asthma [18-20]. Epidemiological data also demonstrated that sleep disturbance is more prevalent in school-aged children with atopic disease, such as hay fever and atopic dermatitis [21,22]. Based on the existing body of knowledge, we hypothesized that SDB may be associated with both the occurrence of asthma and asthma severity. The present study aimed at: I) estimating the prevalence rate of asthma; II) evaluating whether SDB was an independent risk factor for asthma among school-aged children in China; and III) further quantifying the relationship between SDB and prevalence and severity of asthma by conducting a meta-analysis quantitatively incorporating the results from recently published studies.

## Materials and methods

### Multicentric cross-sectional study

#### Study design and subjects

The study design and subjects recruitment protocol have been described in previous studies [15,23,24]. The large national cross-sectional survey was performed in eight Chinese cities in November and December, 2005. A total of 22,478 school-aged children were recruited from six grades of 55 eligible primary schools in 39 districts of eight cities. Out of 22,478 children, 20,672 (91.9%), with a mean age of 9.00 years old (SD = 1.61; ranged from 5.08 to 12.00 years old), returned completed questionnaires.

The protocol of our study was approved by the Ministry of Education of People’s Republic of China and Ethics Committee of Shanghai Jiaotong University, School of Medicine.

### Measures

#### Asthma

Asthma was assessed by addressing the following question to their guardians, “Did pediatricians or pediatric health professionals ever make a diagnosis of asthma for your child?” Answers were coded using a 2-point scale: “0” for no and “1” for yes.

### SDB

The sleep behaviors of subjects were evaluated by Children’s Sleep Habits Questionnaire (CSHQ), which was designed to assess sleep characteristics of pre-school and school-aged children (4 to 12 years old) [23,25]. The sensitivity and reliability of CSHQ have been described in previous studies [15,24].

Three items are included in the subscale regarding the SDB-related signs and symptoms in the CSHQ. The test-retest reliability (ICCs) and the internal consistency (Cronbach’s alpha) of the SDB subscale are 0.76 and 0.42, respectively. The first question is “How often does your child snore loudly during a typical recent week?”. The second question is “How often does your child have snorts and gasps during sleep on a typical recent week?”. And the third one is “How often does your child have stopped or interrupted breathing during sleep on a typical recent week?”.

According to the CSHQ, the question is rated on a 3-point scale: “almost always” for 5 to 7 nights per week; “frequently” for 2 to 4 nights per week; and “occasionally/never” for 0 to 1 night per week. For this study, a SDB-affected subject was defined as an individual SDB problem occurring at least two nights per week.

### Associated factors of asthma

The potential risk factors were conceptually divided into four categories: (1) socioeconomic variables, i.e. family structure, parents’ educational levels and household income; (2) demographic variables, i.e. children’s gender, age and ethnicity; (3) general chronic health problem variables, i.e. overweight/obesity status, history of food/drug allergy and gastro-oesophageal reflux; and (4) respiratory condition, i.e. history of chronic allergic rhinitis diagnosis, frequency of upper respiratory infection and history of hypertrophy of tonsils diagnosis.

### Data analysis

Statistical descriptions including mean, percentage of categorical variables and standard deviation of continuous variables were calculated. Logistic regression was used to assess the association between asthma and symptoms of SDB (Table 1 and 2). All statistical analyses were performed by SPSS version 13.5 (SPSS Inc, Chicago, IL, USA). Two-sided *P* value less than 0.05 was considered as statistical significant.

**Table 1:** **Associated factors regarding asthma by univariate logistical regression models**

**Variables (n, %)**	**Prevalence of asthma n (n %)**	**Univariate regression models**	
		**OR (95% CI)**	***P*** **value**
**Demographic and socioeconomic characteristics**			
Age (years)			
5-6 (2543, 12.3%)	101 (4.0)	1.86 (1.36-2.56)	<.001
7- (3802, 18.4%)	141 (3.7)	1.73 (1.28-2.34	<.001
8- (3839, 18.6%)	128 (3.3)	1.55 (1.15-2.10)	0.005
9- (3798, 18.4%)	131 (3.4)	1.61 (1.19-2.18)	0.002
10- (3724, 18.0%)	134 (3.6)	1.68 (1.24-2.27)	0.001
11- (2943, 14.3%)	64 (2.2)	Ref.	
Gender (%)			
Boys (10227, 49.5%)	434 (4.2)	1.67 (1.44-1.96)	<.001
Girls (10445, 50.5%)	269 (2.6)	Ref.	
Ethnicity			
Han ethnic group (19604, 94.9%)	674 (3.4)	1.40 (0.94-2.08)	0.097
Minority ethnic group (1030, 5.1%)	26 (2.5)	Ref.	
Family income			
<800 (3956, 19.3%)	67 (1.7)	Ref.	
800-2500 (11612, 56.6%)	344 (3.0)	1.77 (1.36-2.31)	<.001
≥2500 (4966, 24.2%)	284 (5.7)	3.52 (2.69-4.61)	<.001
Family structure			0.067
Single parent family (1103, 5.3%)	41 (3.7)	1.17 (0.85-1.63)	0.328
Large family (6565, 31.7%)	249 (3.8)	1.20 (1.02-1.41)	0.024
Nuclear family (13014, 62.9%)	413 (3.2)	Ref.	
Mather’s education level			<.001
Low (5752, 28.2%)	118 (2.1)	Ref.	
Medium (6825, 33.4%)	204 (3.0)	1.47 (1.17-1.85)	0.001
High (7843, 38.4%)	370 (4.7)	2.37 (1.92-2.92)	<.001
Father’s education level			<.001
Low (4940, 23.9%)	109 (2.2)	Ref.	
Medium (7075, 34.2%)	215 (3.0)	1.39 (1.10-1.76)	0.006
High (8647, 41.8%)	379 (4.4)	2.03 (1.64-2.52)	<.001
**General chronic health problems**			
Overweight/obesity			
Obesity (1323, 8.2%)	58 (4.4)	1.44 (1.08-1.91)	0.012
Overweight (2206, 13.6%)	91 (4.1)	1.35 (1.07-1.71)	0.011
Normal (12656, 78.2%)	391 (3.1)	Ref.	
Food/drug allergy			
Yes (1176, 5.7%)	139 (11.8)	4.53 (3.72-5.51)	<.001
No (19588, 94.3%)	565 (2.9)	Ref.	
Gastro-oesophageal reflux			
Yes (159, 0.8%)	19 (11.9)	3.97 (2.44-6.45)	<.001
No (20609, 99.2%)	686 (3.3)	Ref.	
**Respiratory diseases**			
History of chronic allergic rhinitis diagnosis			
Yes (1993, 9.6%)	310 (15.6)	8.65 (7.39-10.12)	<.001
No (18764, 90.4%)	392 (2.1)	Ref.	
Upper respiratory infection			
Frequently (3607 17.4%)	350 (9.7)	5.09 (4.37-5.93)	<.001
Occasionally (17164, 82.6%)	355 (2.1)	Ref.	
History of hypertrophy of tonsils diagnosis			
Yes (2369, 11.4%)	123 (5.2)	1.68 (1.38-2.05)	<.001
No (18404, 88.6%)	582 (3.2)	Ref.	
**Symptoms of sleep disordered breathing**			
Habitual snoring			
Usually/Often (2525, 12.2%)	160 (6.3)	2.20 (1.83-2.64)	<.001
Occasionally/No (18234, 87.8%)	544 (3.0)	Ref.	
Stops breathing			
Usually/Often (282, 1.4%)	24 (8.5)	2.71 (1.77-4.15)	<.001
Occasionally/No (20419, 98.6%)	677 (3.3)	Ref.	
Snorts and gasps			
Usually/Often (640, 3.1%)	80 (12.5)	4.47 (3.49-5.72)	<.001
Occasionally/No (20071, 96.9%)	622 (3.1)	Ref.	

OR, odds ratio; CI, confidence interval.

**Table 2:** **Associations of SDB symptoms with asthma by multivariate logistical regression models**

**Variables**	**Model I**		**Model II**		**Model III**	
	**OR (95% CI)**	***P***	**OR (95% CI)**	***P***	**OR (95% CI)**	***P***
***Habitual snoring***						
Usually/Often	1.97 (1.63-2.38)	<0.001	1.74 (1.39-2.16)	<0.001	1.28 (1.01-1.62)	0.041
Occasionally/No	Ref.		Ref.		Ref.	
***Snorts and gasps***						
Usually/Often	4.45 (3.41-5.82)	<0.001	3.17 (2.26-4.23)	<0.001	1.92 (1.34-2.76)	<0.001
Occasionally/No	Ref.		Ref.		Ref.	

OR, odds ratio; CI, confidence interval.

Model I only adjusted for demographic and socioeconomic characteristics (age, gender, ethnicity, family income, family structure, mother’s education level, and father’s education level).

Model II adjusted for demographic and socioeconomic characteristics and, further, general chronic health problems (overweight/obesity, food/drug allergy, and gastro-oesophageal reflux).

Model III adjusted for demographic and socioeconomic characteristics, general chronic health problem, and respiratory diseases (history of chronic allergic rhinitis diagnosis, upper respiratory infection, and history of hypertrophy of tonsils diagnosis) simultaneously.

### Meta-analysis

#### Literature search

We also conducted a meta-analysis of all published cross-sectional and cohort studies on the correlation between SDB and asthma. Relevant studies published up to June 15, 2014 were retrieved in the MEDLINE, EMBASE, and Chinese National Knowledge Infrastructure (CNKI), without any language restriction, using the following terms: (‘asthma’ or ‘bronchial asthma’) and (‘sleep disorders’ or ‘sleep apnea syndromes’ or ‘sleep-disordered breathing’ or ‘SDB’ or ‘snoring’ or ‘habitual snoring’ or ‘sleep apnea, obstructive’ or ‘OSA’) and (‘child’ or ‘infant’ or ‘child, preschool’ or ‘adolescent’ or ‘adolescent’ or ‘middle aged’ or ‘young adult’ or ‘aged’). Bibliographies of retrieved articles were also reviewed to identify additional eligible articles.

### Data extraction

For each study, we documented information on the first author’s last name, year of publication, country of subject, ethnicities, study design, questionnaire response rate, follow-up period if available, total number of subjects recruited, mean age (or range), symptoms of SDB, and study-specific odd ratios (95% CIs). Ethnicity was categorized as Caucasian, African, Asian, or mixed for studies including subjects of more than one ethnicity. In addition, basic information of each study including country, time and authorship were screened to exclude the duplicated publication.. All data from eligible studies were recorded independently by two authors with a piloted data standardized form and compared afterwards. In cases of conflicting evaluations, minor discrepancies were resolved by a third investigator’s careful full-text reexamination.

### Statistical analysis

Odd ratios (ORs) with corresponding 95% CIs were applied to assess the strength of association of SDB and asthma. Heterogeneity was checked by a Cochran’s *Q*-statistic, a *P*-value less than 0.01 was considered as statistically significant [26]. The *I*^*2*^ test was also used to quantify heterogeneity in terms of percentage (ranging from 0 to 100%) [27]. *P <* 0.01 for *Q*-test or *I*^*2*^ > 50% indicated the existence of heterogeneity between studies. Fixed-effect model (*Mantel-Haenszel* method) was used to pool the data in the existence of between-study heterogeneity; otherwise, a random-effect model (*DerSimonian-Laird* method) should be applied. To evaluate the robustness of the results, a one-way sensitivity analysis was conducted to evaluate the impact of individual study on the pooled results by omitting each study in turn. Egger’s linear regression test and Funnel plot were applied to assess whether the validity of the estimate might be affected by publication bias [28]. The statistical significance of the pooled data was assessed by *Z*-test. All the statistical analyses were conducted using STATA version 12.0 (Stata Corp, College Station, TX, USA).

## Results

### Cross-sectional study

Among all the recruited children, 3.4% of them suffered from lifetime asthma with confirmed diagnosis. The influence of potential contributing factors was also assessed. There was a significant gender difference in the prevalence of asthma (boys: 4.2% vs. girls: 2.6%; *P* < 0.001). In addition, the changing pattern of asthma prevalence in school children manifested a “V” shaped curve, dropping slightly from age 5 to 8, then gradually rising from age 9 to 12.

Table 1 presents the sample characteristics and their associations with asthma diagnosis by univariate analyses. It can be seen that significant differences exist in each demographic variable between asthmatics and non-asthmatics. In univariate regression models, it was revealed that current habitual snoring and OSA may be significant risk factors for asthma.

Multivariate logistic regression was further used to model the association of habitual snoring and OSA with asthma, while adjusting for potential confounders. As shown in Table 2, an initial multivariate analysis was performed to adjust for demographic and socioeconomic variables. The results showed that habitual snoring and OSA are significantly associated with asthma, with odds ratios (ORs) ranging from 1.63 to 5.85. Two additional multivariate models were also conducted, characterized by additional adjustments for asthma-related variables including general chronic health problema and respiratory diseases. Both habitual snoring (OR = 1.28, 95%CI: 1.01-1.62) and OSA (OR = 1.92, 95%CI: 1.34-2.76) remain to be a statistically significant predictor of asthma.

### Meta-analysis

**Figure 1:**
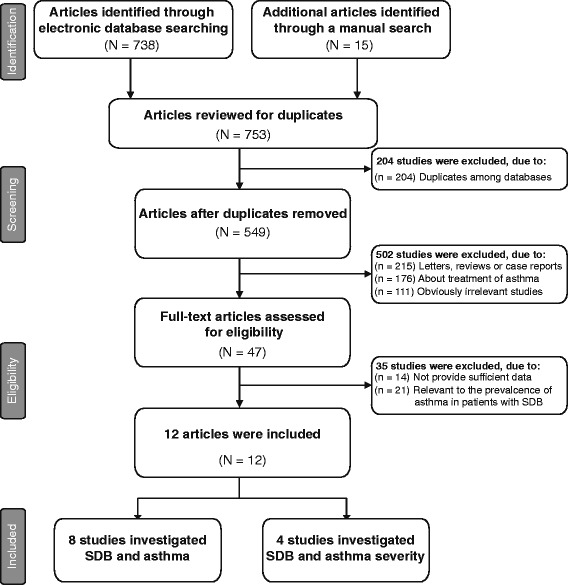
Flow diagram of the selection process of the included/excluded studies in the meta-analysis.

The flow chart showed the process of study selection and exclusion/inclusion criteria in the meta-analysis (Figure 1). 12 studies (not including our cross-sectional study) with 38,766 subjects were included in the analyses [18-20,29-37]. Characteristics of the studies and subjects included were presented in Table 3. Of the 12 included studies, nine were cross-sectional studies, two were cohort studies, and the other one was a case–control study. The sample sizes of included studies range from 60 to 16,191 subjects (mean = 2584). Eight studies investigated whether SDB is an independent risk factor for asthma, while the remaining four studies focused on the correlation between SDB and the severity of asthma.

**Table 3:** **Summary of studies assessing the association of SDB and asthma and its severity risk**

**First author, year**	**Country**	**Ethnicity**	**Study design**	**Response rate**	**Size**	**Male (%)**	**Age (y)**	**Risk factor**	**Outcome**	**OR [95%CI]**
Teodorescu, 2012 [37]	USA	African 6%	Cross-sectional	93%	752	33%	47 ± 14	High OSA risk	Persistent daytime asthma	1.96 [1.31, 2.94]
		Caucasians 91%							Persistent nighttime asthma	1.97 [1.32, 2.94]
		Others 3%						OSA	Persistent daytime asthma	2.08 [1.13, 3.82]
									Persistent nighttime asthma	1.48 [0.82, 2.69]
Ross, 2012 [36]	USA	African/Caucasians	Cross-sectional	54%	108	67.60%	9.1 ± 3.4	SDB	Asthma severity	3.62 [1.26, 5.40]
Lukrafka, 2010 [20]	Brazil	Caucasians	Cross-sectional	93.50%	575	49.80%	Range 12-19	Snoring	Asthma	4.50 [3.30, 6.20]
Teodorescu, 2009 [35]	USA	Caucasians	Cross-sectional	93%	244	39%	46 ± 13	Habitual snoring	Asthma	2.16 [1.13, 4.10]
Kozyrskyj, 2009 [19]	Australia	Caucasians	Cohort study	83.40%	1,999	NA	Range 11-14	Habitual snoring	Persistent asthma (6 yrs)	1.51 [1.04, 2.20]
					1,365				Non-atopic asthma (6 yrs)	2.78 [1.51, 5.09]
					1,693				Persistent asthma (14 yrs)	1.74 [1.05, 2.90]
					1,390				Non-atopic asthma (14 yrs)	2.29 [1.11, 4.71]
Jamrozik, 2009 [34]	Australia	Caucasians	Cohort study	47.30%	1,554	44.5%	Range 20-69	Snore	Newly diagnosed asthma	1.20 [0.90, 1.70]
								Habitual snoring	Newly diagnosed asthma	2.40 [1.40, 4.20]
Karachaliou, 2007 [33]	Greece	Caucasians	Cross-sectional	NA	1,501	59.40%	Range 19-90	Snoring	Asthma	1.01 [0.76, 1.35]
ten Brinke, 2005 [32]	Netherlands	Caucasians	Cross-sectional	46.30%	136	27%	41.5 ± 14.1	OSA	Difficult-to-treat asthma	3.40 [1.20, 10.4]
Ekici, 2005 [31]	Turkey	Caucasians	Cross-sectional	97.70%	10,224	47.50%	44.1 ± 11.6	Snoring	Asthma	1.70 [1.50, 1.80]
Gunnbjornsdottir, 2004 [18]	Denmark	Caucasians	Cross-sectional	74.30%	16,191	47%	39.6 ± 7.1	Snoring	Asthma	1.80 [1.34, 2.42]
Lu, 2003 [30]	Australia	Caucasians	Cross-sectional	61%	974	53%	Range 2-5	Snoring	Asthma	2.00 [1.30, 3.10]
Vir, 1997 [29]	India	Asians	Case–control	NA	60	43.30%	Range 18-28	Snoring	Asthma	2.36 [0.63, 4.92]

OR, odds ratio; CI, confidence interval.

**Figure 2:**
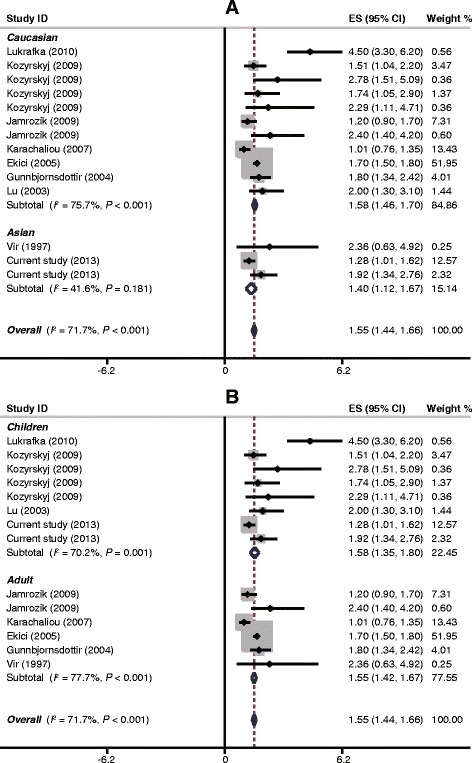
Forest plot of ORs and 95% CIs for the association of SDB with asthma in subgroup analyses based on ethnicity **(A)** and age groups **(B)**.

The result of this meta-analysis was summarized in Table 4. As for SDB and asthma, the random effects model was used since between-study heterogeneity existed (*I*^2^ = 71.7%, *P* < 0.001). The pooled analysis of the eight studies, together with the study presented here, included 59,438 subjects and revealed a significant association between SDB and asthma with OR (95%CI) of 1.55 (1.44-1.66) (Figure 2). Subgroup analysis based on ethnicity showed that the magnitude of the effect was similar in both Caucasians and Asians (OR = 1.58, 95%CI: 1.46-1.70, *P* < 0.001; OR = 1.40, 95%CI: 1.12-1.67, *P* < 0.001) (Figure 2A). In addition, stratified analyses by age group suggested that SDB was significantly correlated with increased risk of asthma in both children and adults (OR = 1.58, 95%CI: 1.35-1.80, *P* < 0.001; OR = 1.55, 95%CI: 1.42-1.67, *P* < 0.001) (Figure 2B). Further subgroup analyses based on type of epidemiological study design indicated that the magnitude of the overall effect was similar in cross-sectional and cohort study (OR = 1.56, 95%CI: 1.45-1.68, *P* < 0.001; OR = 1.46, 95%CI: 1.16-1.75, *P* < 0.001), while the favorable trend disappeared in case–control study (OR = 2.36, 95%CI: 0.21-4.51, *P* = 0.310). As for SDB and asthma severity, since no significant between-study heterogeneity was found (*I*^2^ = 0, *P* = 0.932), the fixed effects model was applied. The pooled analysis of the four studies involving 1,240 patients revealed that the SDB had an OR for severe asthma of 1.92 (95CI: 1.48 to 2.35) (Table 4).

**Table 4:** **Meta-analysis of the association between SDB and asthma and its severity risk**

**Subgroups**	**OR**	**95% CI**	***P*** **value**	***P***_***h***_	***I*** ^***2***^
***Asthma***	1.55	1.44-1.66	<0.001	<0.001	71.70%
*Ethnicity*					
Caucasian	1.58	1.46-1.70	<0.001	<0.001	75.70%
Asian	1.40	1.12-1.67	<0.001	0.150	41.60%
*Age group*					
Children	1.58	1.35-1.80	<0.001	0.001	70.20%
Adults	1.55	1.42-1.67	<0.001	<0.001	77.70%
*Study design*					
Cross-sectional	1.56	1.45-1.68	<0.001	<0.001	84.4%
Cohort study	1.46	1.16-1.75	<0.001	0.249	247%
Case–control	2.36	0.21-4.51	0.310	n.a.	n.a.
***Asthma severity***	1.92	1.48-2.35	<0.001	0.932	0.00%

OR, odds ratio; CI, confidence interval; *P*_h_, *P*-value of heterogeneity; n.a., not available.

In this meta-analysis, sensitivity analyses were performed to evaluate the impact of individual study on the overall results. The pooled ORs of SDB with asthma and asthma severity were not affected by the results of any individual study, suggesting the statistically robust results of this meta-analysis. Furthermore, Begg’s funnel plot and Egger’s linear regression test were performed on the metadata to evaluate publication bias. Egger's test and Funnel plot indicated no publication bias in this meta-analysis (t = 0.68, *P* = 0.511; t = 1.99, *P* = 0.103).

## Discussion

Up to now, this is the largest epidemiological study exploring the relationship between SDB and childhood asthma (n = 20,672). The reported asthma cases accounted for 3.4% of our study population, which was higher prevalent than that previously reported in Jinan districts, China (sample aged 0–14 years, 0.7%) [38]. In addition, this study identified a relatively high prevalence of habitual snoring as well (12.2%), compared to 10.9% in Hong Kong children aged 6–12 years [28], 10.0% in French children aged 5 to 6 years [12], and 11.4% in British children aged 4 to 7 years [39].

In this context, we have assembled the first national school-based survey in 5–12 years children by parent-completed questionnaire to assess separately the influence of habitual snoring and OSA on asthma. In the current study, both OSA and habitual snoring were independently associated with onset of asthma after adjusting for traditional asthma risk factors. Although previous studies of stable nocturnal asthma had concluded that impaired quality of sleep, with disturbed sleep during the night, early morning awakenings and daytime sleepiness is common in asthmatic patients, these studies only focused on the effect of asthma on sleep behaviors [40,41]. In recent years, an increased prevalence of sleep-related disorders, such as snoring, self-reported apnea, difficulty in inducing and maintaining sleep, and daytime sleepiness in asthmatic subjects has been reported [18,30,42]. In addition, other studies also have reported patients with SDB are more vulnerable to asthma [19,20]. All these studies, together with our cross-sectional study and meta-analysis, support the hypothesis that OSA and habitual snoring are independent risk factors for asthma.

Asthma is a diffuse airway inflammation involving small and large airways and habitual snoring. It is presumably associated with inflammation of upper airways, which could also result in atopy. It is not surprising that they may coexist in the same individual. Although the clear mechanisms underlying these associations remain to be explored, there are several mechanisms how SDB might be linked with asthma. SDB is associated with elevation of pro-inflammatory cytokines, excessive daytime sleepiness (EDS), increased leptin levels, and reduced adiponectin levels [43]. The two adipokines, produced by the adipose tissue that has important pro-inflammatory properties, are correlated with the occurrence of asthma [44]. Moreover, it is possible that the correlation between asthma and SDB might partially be due to the fact that the presence of upper airway inflammation is common in asthma patients.

As discussed above, SDB was also shown to be associated with the onset of asthma. In this meta-analysis, we further examined the potential interaction between SDB and asthma severity. A clear relationship between reported snoring and severe asthma was identified even when likely confounding factors were adjusted, suggesting that patients who have difficulty achieving adequate asthma control should be screened for SDB. The mechanisms through which SDB worsens asthma still remain largely unclear. Our finding has biological plausibility since Irwin *et al*. observed that tumor necrosis factor (TNF-α), one of the pro-inflammatory cytokines, participate in sleep control and elevate in sleep-deprived adults [45]. TNF-α has been reported as a marker of 'systemic' inflammation in adults with severe asthmatics [46]; neutrophilic airway inflammation has been observed in children with OSA [47]. In addition, there is evidence that the treatment of snoring and OSA with nasal-continuous positive airway pressure (nCPAP) can lead to improved control of asthma both during the night and while awake [48].

Several limitations of our study should be addressed. Firstly, this study is questionnaire-based. No objective measurement, such as attended polysomnography, was performed to diagnose SDB. It may cause measurement bias. Meanwhile, it is impossible to determine whether the subjects reporting asthma actually were asthmatics due to the lack of a gold standard. Another drawback was the direction of the cause-effect relationship between SDB and asthma or whether there is a true cause-effect relationship, due to the limitation of cross-sectional data in drawing causality conclusion. The statistical pooling of independent studies also has some disadvantages. Last, the relationship between SDB and asthma severity was not evaluated in our cross-sectional study due to the limited information obtained from the questionnaire. Firstly, meta-analysis cannot correct the limitations of primary research. Secondly, most of included studies for meta-analysis were performed in Caucasians, which restricts the findings to be generalized to other populations in different ethnicities. Finally, although these estimations were based on individual adjusted ORs, and we adjusted our analysis for a broad range of potential confounding factors, there is still a chance of unmeasured or residual confounding. Despite the limitations described above, our study had a large sample size that may increase the validity of results. Further, the meta-analysis summarized the previous studies and our study, which could provide insight into the association between SDB and asthma stratified by ethnicity and age. Up to now, this is the first national study to investigate whether SDB was an independent risk factor for asthma in school-aged children.

In conclusion, our large epidemiological study, for the first time, examined the association of asthma with habitual snoring and OSA among Chinese children. Our findings provide new evidence to support current asthma guidelines. The pooled data from this large cross-sectional study and meta-analysis will enrich our knowledge on the relationship between SDB (habitual snoring and OSA) and asthma. Further studies should focus on elucidating the underlying mechanism, such as how SDB is associated with asthma.

## Abbreviations

SDB: Sleep-disordered breathing
OSA: Abstructive sleep apnea
OR: Odd ratio
